# High-throughput dielectrophoretic filtration of sub-micron and micro particles in macroscopic porous materials

**DOI:** 10.1007/s00216-020-02557-0

**Published:** 2020-03-21

**Authors:** Malte Lorenz, Daniel Malangré, Fei Du, Michael Baune, Jorg Thöming, Georg R. Pesch

**Affiliations:** 1 Chemical Process Engineering (CVT), University of Bremen, Leobener Str. 6, 28359 Bremen, Germany; 2 Center of Applied Space Technology and Microgravity (ZARM), University of Bremen, Am Fallturm 2, 28359 Bremen, Germany; 3 MAPEX Center for Materials and Processes, University of Bremen, 330 440, 28334 Bremen, Germany

**Keywords:** Dielectrophoresis, Micron and sub-micron particle separation, Material-selective particle filtration, Open porous ceramic microstructures

## Abstract

State-of-the-art dielectrophoretic (DEP) separation techniques provide unique properties to separate particles from a liquid or particles with different properties such as material or morphology from each other. Such separators do not operate at throughput that is sufficient for a vast fraction of separation tasks. This limitation exists because high electric field gradients are required to drive the separation which are generated by electrode microstructures that limit the maximum channel size. Here, we investigate DEP filtration, a technique that uses open porous microstructures instead of microfluidic devices to easily increase the filter cross section and, therefore, also the processable throughput by several orders of magnitude. Previously, we used simple microfluidic porous structures to derive design rules predicting the influence of key parameters on DEP filtration in real complex porous filters. Here, we study in depth DEP filtration in microporous ceramics and underpin the previously postulated dependencies by a broad parameter study (Lorenz et al., 2019). We will further verify our previous claim that the main separation mechanism is indeed positive DEP trapping by showing that we can switch from positive to negative DEP trapping when we increase the electric conductivity of the suspension. Two clearly separated trapping mechanisms (positive and negative DEP trapping) at different conductivities can be observed, and the transition between them matches theoretical predictions. This lays the foundation for selective particle trapping, and the results are a major step towards DEP filtration at high throughput to solve existing separation problems such as scrap recovery or cell separation in liquid biopsy.

**Electronic supplementary material:**

The online version of this article (10.1007/s00216-020-02557-0) contains supplementary material, which is available to authorized users.

**Figure:**
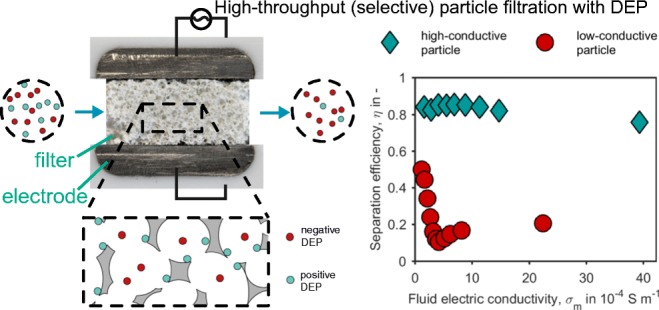
Graphical abstract

## Introduction

Separation of micron and sub-micron particles from liquid media or according to their properties is essential for a wide variety of fields. It is a key for (bio-)analytics and medical diagnostics [1], for example for cell separation in liquid biopsy, as well as product purification [2]; for recovery and mining of valuable materials [3–5]; or to increase the sustainability and cost efficiency of industrial processes. For example, during the recovery of precious materials from electronic waste, one of the first steps is to shred down the electronic waste to small pieces. Then, standard physical separation processes for material recovery can be used that are based on, for instance, differences in density or magnetism. Noble metals are concentrated in the fine dust that is produced as a by-product during milling, and 10% to 35% of the total amount of noble metals are currently lost because of inefficient separation processes for this fraction [5, 6]. A separation technique to recover these valuable particles from the dust would allow to mine otherwise lost materials from waste. Currently available separation techniques, such as deep-bed filtration and centrifugation, can be used to separate particle systems according to size or density. However, at small particle scales (of the dimension of cells or fine dust), density separation fails as the weight differences become negligible and size exclusion mechanisms require high pressure differences to achieve significant throughputs. Thus, different approaches are required.

Dielectrophoresis (DEP) is an electrokinetic phenomenon that can be exploited for highly selective separation techniques [7]. It was, for example, applied to separate target cells/particles against millions of background particles [8], live and dead cells [9], blood cells according to type, and circulating tumor cells from whole blood [1, 10–12]. However, most DEP-based separators require microfluidic devices and lack the capability to process sufficiently high throughputs to handle separation tasks at industrial or preparative scale. The reason for why these separators offer high selectivity but limited throughput lies in the inherent physics of DEP.

DEP-based separation techniques utilize spatially non-uniform electric fields to move polarizable particles [13]. The DEP force depends on the particle volume, its relative polarizability, and the spatial change of the electric field. Using the point-dipole approximation, the DEP force 〈**F**_DEP_〉 can be expressed as

1 $$ \left\langle {\mathbf{F}}_{\mathrm{DEP}}\right\rangle =\frac{1}{4}\pi {d}_{\mathrm{P}}^3\operatorname{Re}\left[K\right]\nabla {\left|{\mathbf{E}}_{\mathrm{RMS}}\right|}^2 $$

with the del operator ∇ = (*∂*/*∂x*, *∂*/*∂y*, *∂*/*∂z*), which gives the gradient of a scalar field, the particle diameter (*d*_P_), the real part of the complex Clausius-Mossotti (CM) factor (Re[*K*]), and the root-mean-square electric field vector (**E**_RMS_). Re[*K*] describes the relative electric polarizability of a particle with respect to the surrounding medium and represents the reason why DEP can be applied for selective particle separation according to dielectric properties.The CM factor depends on the complex permittivities of the particle, ${\overset{\sim }{\varepsilon}}_{\mathrm{P}}$, and the medium, in which the particle is suspended ${\overset{\sim }{\varepsilon}}_{\mathrm{m}}$, and its real part is given by

2 $$ \operatorname{Re}\left[K\right]=\operatorname{Re}\left[\frac{{\overset{\sim }{\varepsilon}}_{\mathrm{P}}-{\overset{\sim }{\varepsilon}}_{\mathrm{m}}}{{\overset{\sim }{\varepsilon}}_{\mathrm{P}}+2{\overset{\sim }{\varepsilon}}_{\mathrm{m}}}\right]. $$

The complex permittivity describes the frequency (*ω*)-dependent polarizability of a material and is dependent on the material’s permittivity (*ε*) and conductivity (*σ*), where $\overset{\sim }{\varepsilon }=\varepsilon +j\frac{\sigma }{\omega }$. At low field frequencies, particle polarization is only dependent on the electrical conductivity of the particle (*σ*_P_) and the surrounding medium (*σ*_m_) and the real part of the CM factor becomes

3 $$ \operatorname{Re}\left[K\right]=\frac{\sigma_{\mathrm{P}}-{\sigma}_{\mathrm{m}}}{\sigma_{\mathrm{P}}+2{\sigma}_{\mathrm{m}}} $$

because the majority of the charge that causes polarization is transferred by conduction [14]. At high frequencies, this mechanism changes because the time for charges to align with the field due to conduction is too short; then, charge separation occurs due to molecular polarization mechanisms (which is expressed through the permittivities of the particle and medium). Re[*K*] can take values between 1 and − 0.5, and the sign dictates if particles will move along or against the electric field gradient; this allows to move particles of different polarizabilities (different dielectric signatures) to opposite directions in the field gradient. If the particle is more polarizable than the surrounding medium, the CM factor will be positive, and the particle will experience positive dielectrophoresis (pDEP) resulting in a force pointing towards higher electric field regions (with the field gradient). On the other hand, if a particle is less polarizable, the CM factor is negative, the particle will experience negative dielectrophoresis (nDEP), and the acting force points towards low electric field regions (against the field gradient). There are numerous approaches to use this effect for selective particle separation [7, 15].

Most DEP-based studies show high selectivity at the expense of low throughputs in the range of mL h^−1^ that are only suited for handling very small samples. While for many other separation techniques, surface interactions on the molecular scale are the main mechanism, DEP relies on the action of an inhomogeneous electric field on a particle. The DEP force depends on the *square* of the electric field gradient (Eq. (1)); hence, the force acting on a particle decreases exponentially with distance from the asymmetrical electrodes (in case of electrode-based DEP devices) or from the insulating structures (in case of electrode-less DEP devices). As a consequence, in traditional DEP devices, the separation efficiency is coupled to the device dimensions: Small distances between the electrodes or insulators and additionally small channel sizes are required to generate sufficient electric field gradients [14]. In microfluidic devices, the channel height is further restricted due to the fabrication, which limits the cross section and therefore the throughput of these devices.

**Fig. 1:**
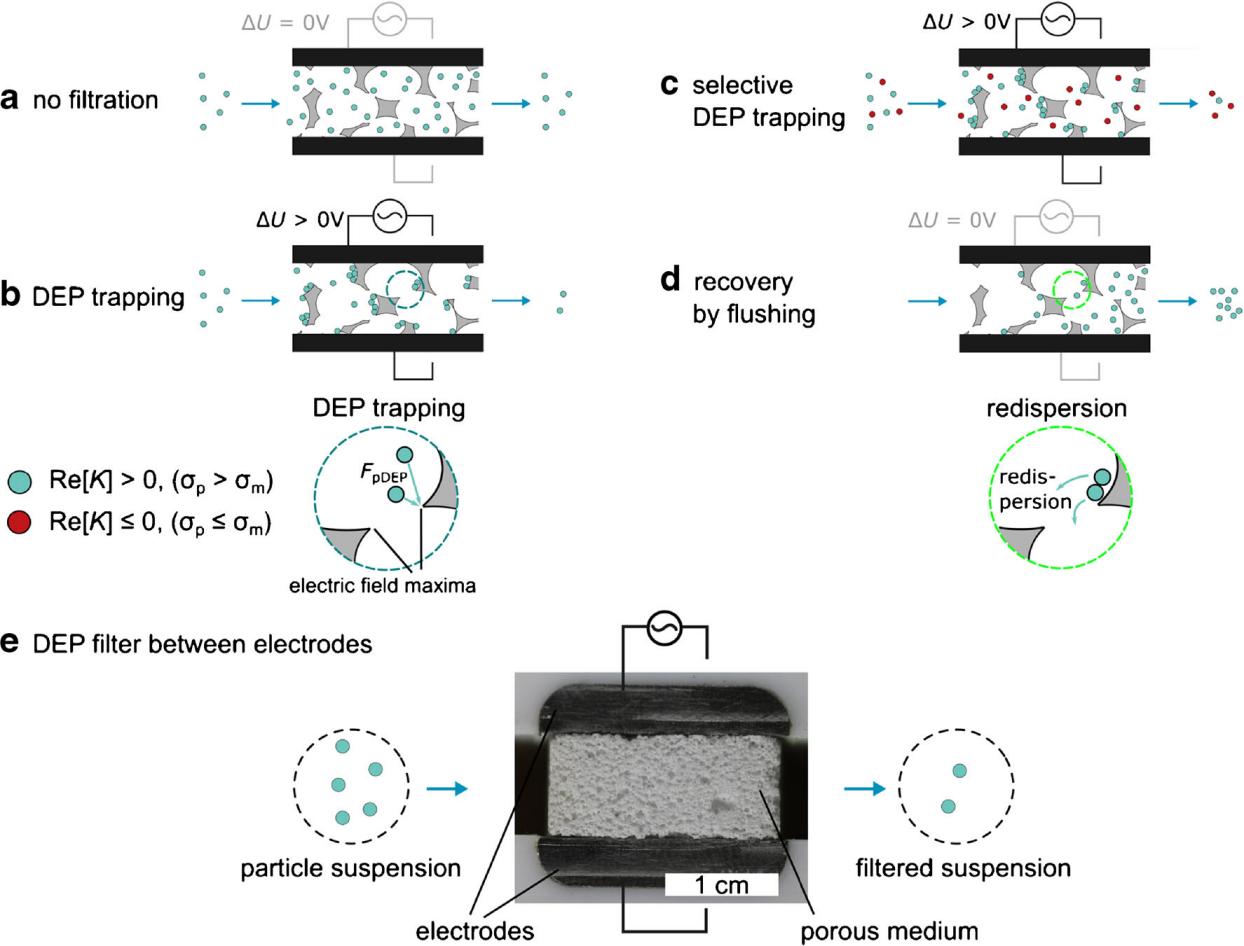
Dielectrophoretic filtration allows switchable particle separation from a liquid or according to particle properties, for example conductivity or size. Particles are pumped through a porous filter medium that is sandwiched between two electrodes. The pore windows of the filter are much bigger than the particle size, and thus, the particles will penetrate the filter (follow the flow) when no electric field is applied (**a**). An electric field will be locally disturbed by the porous filter resulting in a vast number of electric field maxima and dielectrophoretic particle motion. Particles that are more polarizable (pDEP) than the surrounding medium (blue) are pulled towards the electric field maxima at the filter wall where they are trapped (**b**). Particles, equal (no DEP) or less polarizable (nDEP) than the medium (red), are not affected or pushed away from the field maxima and thus pass through the filter because they remain in regions of dominant fluid flow. This allows to selectively trap particles from particle mixtures (**c**). When the electric field is switched off, trapped particles are redispersed and can be recovered at higher concentration by flushing the filter (**d**). The DEP filter (between the electrodes) that was used for experimental study is shown on the bottom (**e**). The filter has a depth of 2.8 cm, and the particle suspension flows from left to right. An image of the whole filter cell is shown in Fig. 2 a

Bridging the gap from low- to high-throughput dielectrophoretic applications is an unexplored challenge our group focuses on [16–19]. One way to increase the throughput is to increase the device’s cross section as it is done in dielectrophoretic filtration. In this approach, the electric field gradients are not generated by a highly asymmetrical and small electrode design, but porous microstructures are used to disturb an originally homogeneous electric field that is generated by two macroscopic electrodes. The electric field gradient is therefore mainly dependent on the design of the porous microstructure [19, 20], whereas the electrode distance can be increased by several orders of magnitude (centimeter and above) as long as the voltage is increased by the same factor. The dielectrophoretic filtration technique uses the inhomogeneous electric fields generated in such microstructures to trap particles from a pumped input suspension (Fig. 1). Since the electric field maxima are primarily located at the interface between fluid and microstructure, particles that experience positive DEP are pulled out of the fast-moving bulk fluid flow towards these interfaces where they are trapped. Particles that experience negative DEP are pushed away from the interfaces into the fast-moving fluid flow and carried through the structure. The porous microstructure can therefore work as an electrically switchable filter that retains, for example, particles that are more polarizable (conductive) than the surrounding fluid (that experience positive DEP) but is permeable to particles of lower polarizability (conductivity) than the surrounding fluid (that experience negative DEP).

The few existing studies from other groups on DEP filtration show manifold-increased throughputs compared to microfluidic approaches. For example, DEP filtration was used to separate yeast cells from water at moderate flow rates of 6 mL min^−1^ [21–23]. In older studies, it was already shown that metallic ceramic and plastic particles could be filtered dielectrophoretically from nonconductive liquids at flow rates of 1 L min^−1^ [24, 25]. Wakeman and Butt [26] filtered air conditioning (AC) test dust and PVC particles from oil and achieved filter efficiencies up to 60% at flow rates of 5 L min^−1^. All of those studies worked phenomenologically and focused on specific separation tasks. In order to understand and prospectively design separation processes and devices, knowledge about key parameters that influence the trapping in porous structures is required.

With this aim, we recently derived design rules in a proof-of-principle study using a microfluidic chip and applied them in a macroscopic setup [17]. In the macroscopic setup, we achieved throughputs of almost 10 mL min^−1^ retaining up to 90% of baker’s yeast cells from an aqueous suspension and found that our design rules predicted the trapping very well. However, we cannot imply a general validity from this rather specific experiment. A broader experimental investigation is required to underpin the validity of the derived design rules for arbitrary porous structures. Further, the design rules presume that the major mechanism behind particle trapping in porous structures is indeed positive DEP. Since we cannot rule out that trapping is driven by other phenomena, such as vortices from nonlinear electrokinetics, as described by Wang et al. [27], this assumption needs verification. In the present experimental study, we treat both these points by (1) scrutinizing this process and the underlying design rules further using model polystyrene (PS) particles and open porous ceramic filters and (2) showing for the first time that particle trapping is primarily driven by DEP. Further, we focus on the potential that DEP filtration provides (Fig. 1a–d), regarding switchable particle filtration from liquid, selective particle filtration based on their relative polarizability, and recovery of the trapped particles. Experiments were performed with model PS and graphite particles and open porous ceramic filters that were sandwiched between two plate electrodes (Fig. 1e).

## Materials and methods

### Particles and particle suspensions

All experiments, except the ones about electrical conductivity-selective particle separation, were conducted using the same particle suspension. Monodisperse-carboxylated PS particles with a diameter of 0.5 μm (Polysciences Fluoresbrite YG Carboxylate Microspheres 0.5 μm, coefficient of variation as measured by the manufacturer 3%) were diluted in ultrapure water that had been degassed under reduced pressure (80 mbar) to a concentration of 2.2 × 10^6^ particles cm^−3^. A small amount of Tween 20 (0.01 vol%) was added to reduce particle adsorption to the filter. The electrical conductivity was adjusted with KCl to 1.2 × 10^−4^ S m^−1^. For the experiments on electrical conductivity-selective particle separation, we used the same suspension but particles with a diameter of 4.5 μm (Polysciences Fluoresbrite, YG Carboxylate Microspheres 4.5 μm, CV 7%) at a concentration of 2 × 10^4^ particles cm^−3^. Graphite particles were taken from a graphite water dispersion (Graph Aqua, AMG Graphite GK) with an average diameter of about 3 μm (manufacturer’s information). Fifty microliters of the graphite dispersion was diluted in 500 mL aqueous suspension. The fluid electrical conductivity of the resulting suspension for these experiments was adjusted by adding KCl.

### Filtration setup

**Fig. 2:**
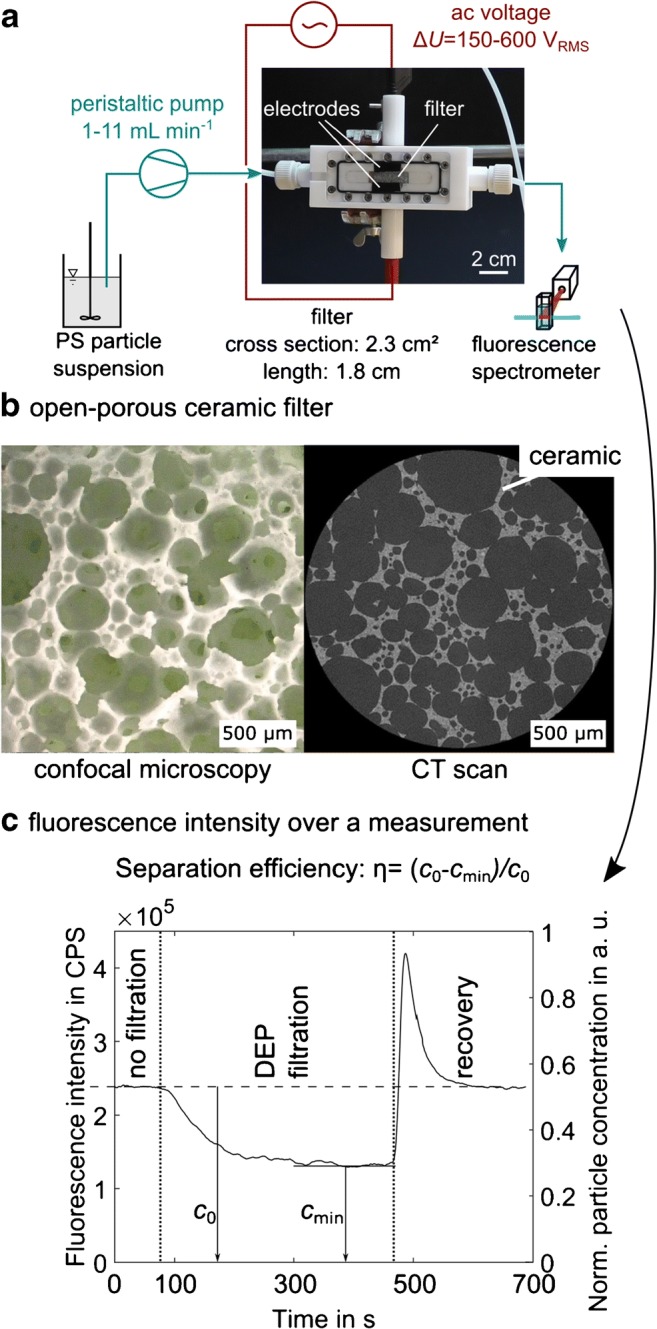
Scheme of the experimental setup with a picture of the filter cell (**a**). Polystyrene particles were constantly pumped with flow rates from 1 to 11 mL min^−1^ through the porous filter that is sandwiched in between two stainless steel electrodes. The electrodes that are placed parallel to each other with a distance of 8 mm were supplied with ac voltages of 150 V_RMS_ to 600 V_RMS_ at a frequency between 1 and 15 kHz. The filter has a width of 8 mm, a height of 29 mm, and a length (in flow direction) of 18 mm. (**b**) Images of the filter structure (confocal microscopy and computed tomography scan). The particle concentration at the outlet of the filter is measured by fluorescence spectroscopy. The fluorescence signal is linearly dependent on the particle concentration, and the separation efficiency was defined as *η* = (*c*_0_ − *c*_min_) / *c*_0_. Here, *c*_0_ is the normalized particle concentration when no electric field is applied, and *c*_min_ is the minimum normalized concentration detected when the field is applied. An exemplary plot of the fluorescence intensity in counts per second (CPS)/normalized particle concentration for one measurement cycle is shown in plot (**c**). In this plot, the flow rate during recovery was 5.5 times as high as the flow rate during DEP filtration and the particle recovery rate for this specific experiment was 82%

A schematic of the experimental setup is shown in Fig. 2 a. The volumetric flow of the suspension through the filter cell was controlled between 1 and 11 mL min^−1^ by a peristaltic pump (REGLO Analog, Ismatec). A picture of the filter cell is shown in Fig. 1 e. It consists of a tapered inlet and outlet (to prevent particle accumulation) and the porous filter that is tightly sandwiched in between two stainless steel plate electrodes. A sinusoidal ac voltage between 150 and 600 V_RMS_ at 1 kHz to 15 kHz was applied across the distance of 8 mm between the electrodes (using a TREK PZD700A power amplifier in combination with a Hameg HM8131 function generator) generating an electric field inside the filter medium perpendicular to the filtrate flow. The power required for DEP in the filter is significantly higher than the power required in microfluidic DEP devices (difference in dimension) which limited the output frequency of our current amplifier to 15 kHz. The replaceable porous filter had a cross section of 8 mm × 29 mm, and the filter depth in flow direction was 18 mm. The particle concentration after the filter was determined by using a FluoroMax 4 fluorescence spectrometer (Horiba) and a quartz flow-through cuvette (176.762-QS, Hellma). This allowed us to online detect the fluorescence intensity signals of the filtrate. At the particle concentrations we used in this study, the fluorescence intensity signal is linearly dependent on the particle concentration (as validated by preliminary experiments, not shown), allowing highly accurate particle concentration measurements. The fluorescently labeled particles were excited at a wavelength of 441 nm, and emission was detected at 486 nm matching excitation and emission maxima of the particles. The concentration of the graphite particles that were not labeled by a fluorescent dye was measured by detecting the reflection intensity (excitation and detection wavelength was 600 nm). Again, calibration measurements were done to verify that the reflection intensity was linearly dependent on the graphite particle concentration.

### Porous filter material

The open porous alumina-mullite ceramics were produced by direct foaming. Information about their fabrication is provided in the Electronic Supplementary Material (ESM; section A). Figure 2 b shows two exemplary images of the pore structure (a confocal microscope image and one slice from a computed tomography scan). The structure shows spherical pores that are highly connected (highly open porous) by numerous pore windows with sharp thin edges. We used filters with porosities of about 83% and four different structure sizes. Hydraulic pore diameter, volume-weighted median pore diameter, and area-weighted median pore window diameter were determined from computer tomography data by using MATLAB and the watershed algorithm implemented in the DIPimage package, version 2.9. A detailed description of the method is provided in the ESM (section B, Fig. S1). Additionally, to allow comparison with previous studies [21, 28], a packed bed of glass beads (350 μm diameter) was used as porous material. The packed bed had the same dimensions as the ceramic filter and was kept in place by two porous sintered glass filters (pore size 160 μm to 250 μm) that sealed the 8 mm × 29 mm gaps at the sides of fluid inflow and outflow for the glass beads. The structural sizes are listed in Table 1.

**Table 1:** Hydraulic pore diameter (*d*_h_), volume-weighted median pore diameter (*d*_p,3_), and area-weighted median pore window diameter (*d*_w,2_) of the filters

Filter	*d*_h_ (μm)	*d*_p,3_ (μm)	*d*_w,2_ (μm)
maliS	222	320	178
maliM	255	394	178
maliL	429	617	226
maliXL	480	642	272
Glass beads 350 μm	156		

### Experimental procedure

All experiments were done with ceramic filters/glass beads that were used multiple times. To provide constant conditions for each experiment, the setup was flushed with ethanol prior to experiments, to clean the setup and wash out particles and air. Afterwards, the setup was flushed with degassed and deionized water to wash out the ethanol. Subsequently, the particle suspension was pumped into the setup. To guarantee a constant particle concentration at the inlet, the particle suspension was permanently stirred. Each measurement was performed in three steps.

1. Start-up phase: The volumetric flow rate (*Q*) was set but no electric field was applied, and the particle concentration in the filtrate without dielectrophoretic trapping was determined (*c*_0_) (Fig. 2c). Since *c*_0_ was determined at the filter outlet, it already contains mechanical trapping (see below).
2. DEP trapping phase: The electric field was applied. In response, the particle concentration decreased until it reached a constant minimum (*c*_min_). *c*_min_ is reached with a delay because the initially measured filtrate only traveled parts of the filter and had a shorter retention time in it.
3. Recovery phase: The field was switched off, and the filter was flushed at the possible highest flow rate, *Q*_rec_ = 11 mL min^−1^. Flushing was performed with the particle suspension. The particle concentration peaked and then fell slowly back to *c*_0_. We found that the time to reach this minimum concentration was independent of DEP-related parameters. It agreed with the particle retention time in the setup that was determined as the time between injecting a concentration jump at the filter inlet until a constant outlet concentration was reached.

#### Mechanical trapping

A small number of particles is filtered without electric field. They are transported to the inner surface of the filter by sedimentation, interception, inertia, and hydrodynamic forces [29] and immobilized at the inner surface due to electrostatic and van der Waals forces [30, 31] as well as wedging or straining [32]. For each filter, the amount of mechanically trapped particles was determined by comparing the particle concentration at the filter outlet (with no electric field applied) to the concentration at the filter inlet.

#### Separation efficiency

The separation efficiency (*η*) was defined as the number of particles that was trapped in the filter by the electric field normalized by the number of particles that would exit the filter without electric field, *η* = (*c*_0_ − *c*_min_) / *c*_0_. It is not dependent on mechanical trapping because *c*_0_ already accounts for mechanical trapping effects.

#### Particle recovery rate

The particle recovery rate (*R*) describes the proportion of dielectrophoretically trapped particles that can be recovered when the electric field is switched off and the filter flushed. The amount of trapped particles was determined by multiplying the temporal integral between *c*_0_ and filter outlet concentration over the time of DEP trapping (*A*_trap_) (Fig. 6) with the applied volumetric flow rate (*Q*). The amount of recovered particles was calculated analogously for the time of recovery. Thus, the particle recovery rate was calculated by *R* = *A*_rec_ × *Q*_rec_ / (*A*_trap_ × *Q*_trap_). The volumetric flow rate for recovering the particles from the filter (*Q*_rec_) was always set to 11 mL min^−1^.

#### Filter capacity

In this study, filter capacity is described by the number of particles that can be trapped in the filter until the separation efficiency decreases to 60.6% of the initial (i.e., maximum) trapping efficiency to describe the capacity. This separation efficiency is reached after *τ*/2, with the relaxation time (*τ*) after that the trapping efficiency decreases to 36.8% of the initial value.

## Results and discussion

In our previous study [17], we combined simulation and experiments in microchannels using highly simplified model filter structures to derive design rules for high-throughput DEP filtration in complex macroscopic filter structures. We predicted that the separation efficiency in porous structures, in general, is a function of $\overline{x}={\left(\Delta U\right)}^2{Q}^{-1}{d}_{\mathrm{P}}^2$, with the applied voltage (Δ*U*), the volumetric flow rate (*Q*), and the particle diameter (*d*_p_). As an outlook, we showed in a single experiment trapping of baker’s yeast in a macroscopic ceramic filter structure and found that the predicted design rules and trapping results matched well. The good match between DEP simulation and filtration in a real random and inhomogeneous macroscopic structure was by no means obvious, since fluid dynamics and electrokinetics are highly complex in such systems. It also remained an open question, if the main trapping is indeed DEP driven or due to other DEP-related, nonlinear electrokinetic effects that were reported in macroscopic structures [33]. In this study, we will focus on an experimental investigation of DEP filtration in macroscopic structures and

1. confirm the validity of the postulated design rules [17] by an expanded parameter study focusing on the influence of flow rate, voltage, and the filter structure;
2. verify the hypothesis that DEP drives particle trapping by showing the transition from pDEP to nDEP trapping due to the change of the suspension’s electric conductivity;
3. show that pDEP and nDEP result in different trapping efficiencies which is a major step for advancing the technique towards high-throughput selective separation of particles with different dielectric properties; and
4. attend to filter capacity and particle recovery.

### Influence of volumetric flow rate, applied voltage, and filter structure size

**Fig. 3:**
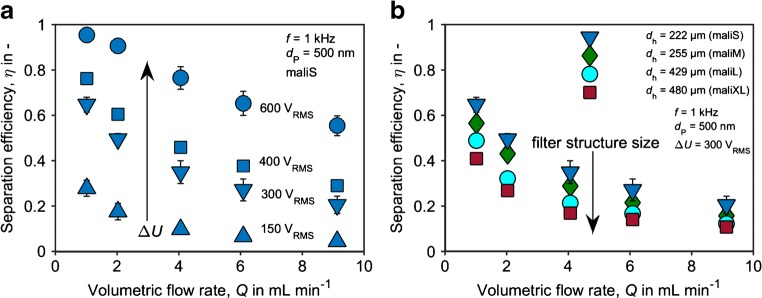
Separation efficiency as a function of volumetric flow rate and the applied voltage (**a**) and filter structure size (**b**). Each data point represents the average of three measurements. The corresponding standard deviations are given by the error bars

For the parametric study, we used fluorescent 500-nm polystyrene beads, which are an order of magnitude smaller than the formerly used baker’s yeast. At the chosen medium conductivity and frequency, the particles show positive DEP (see ESM, section E, for a discussion of polystyrene particle polarization). Mechanical trapping of the 500-nm PS particles was 2% for all flow rates of this study between 1 and 11 mL min^−1^ and, compared to DEP-driven particle retention, low. At a given filter structure and a given particle suspension, the DEP separation efficiency increases with decreasing volumetric flow rate and increasing applied voltage (Fig. 3a). This meets expectations because a particle is trapped when it is pulled into a “trapping zone” (i.e., an electric field maximum) where the DEP force dominates over the drag force exerted by the fluid onto the particle. The probability that a particle is trapped (represented by the separation efficiency) is therefore dependent on these two competing forces. The DEP force (Eq. (1)), and thus, the DEP migration velocity of the particles correlates with the applied voltage squared. The volumetric flow rate, on the other hand, correlates linearly with the superficial velocity and hence the time that a particle has to migrate into a trapping zone where it is trapped.

While the fluid flow and electric field in the randomized ceramic filter are too diverse to investigate the trapping conditions in the filter in detail, our results still show that separation increases with decreasing filter structure size (Fig. 3b), when the other parameters are kept constant. We chose to use the hydraulic pore diameter (*d*_h_) as the characteristic structural size. For a good approximation, we can assume that the whole structure is equally scaled down in size (smaller but more pores with identical shape). In this case, the volume averaged ∇|**E**_RMS_|^2^ in the filter void volume is inversely dependent on the scaling (ESM, section C). Thus, for particles that are randomly distributed in the filter, the effective average DEP force is inversely proportional to the filter’s structural size (*s*) (which is proportional to window and pore window diameter) and the square of the applied voltage (∆*U*). We will see below that the trapping efficiency is also a function of *s*^−1^∆*U*^2^.

### Scaling laws in porous filter materials

**Fig. 4:**
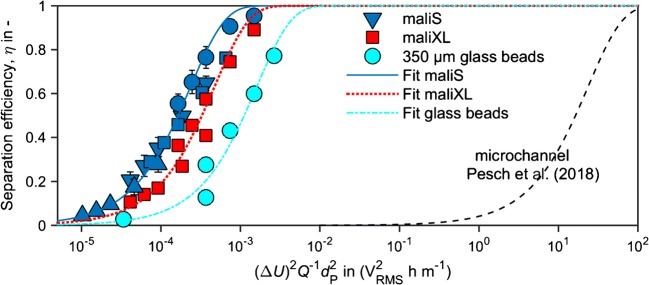
Separation efficiency (*η*) as a function of $\overline{x}={\left(\Delta U\right)}^2{Q}^{-1}{d}_{\mathrm{P}}^2$ for the finest and the coarsest ceramic filters and 350-μm glass beads, varied voltage (Δ*U* = 150–600 V_RMS_), and volumetric flow rate (*Q* = 1–9 mL min^−1^) compared to simulated and experimentally validated results of Pesch et al. [17] (dashed line). The results for the filter structure maliS (blue) are the ones shown in Fig. 3 a. Fitting was done using *η* = (1 − exp($\overline{x}/C$)) resulting in *C* = − 24.6 × 10^−2^ V_RMS_ h^−1^ m^−1^ (maliS), *C* = − 49.2 × 10^−2^ V_RMS_ h^−1^ m^−1^ (maliXL), and *C* = − 3.2 V_RMS_ h^−1^ m^−1^ (350 μm glass beads)

When the results from Fig. 3 are plotted against $\overline{x}={\left(\Delta U\right)}^2{Q}^{-1}{d}_{\mathrm{P}}^2$ (Fig. 4), they confirm the predicted scaling from our previous study [17]. The trapping efficiency of all filters can be described by *η* = (1 − exp($\overline{x}/C$)), with *C* being a fitting coefficient that we used to match experimental results. As already discussed in [17], in comparison to the separation efficiency achieved in microchannels (dashed line), the results in the porous filter are shifted by 5 orders of magnitude to the left. Consequently, in the filter, $\overline{x}$ (which could be interpreted as the cost for operating the process) can be 5 orders of magnitude lower than that in the microchannel, to achieve the same separation efficiency. In other words, in the filter, we achieve the same separation efficiency at the same applied field strength and particle diameter but at a 5-orders-of-magnitude higher volumetric flow rate. We used a fitting parameter of *C* = − 24.6 × 10^−2^ V_RMS_ h^−1^ m^−1^ for the fit through the finest porous filter (maliS). Replacing the finest porous filter by the coarsest (maliXL) led to a horizontal shift by a factor of 2. Since pore and pore window diameters indicate that the structural size (*s*) of maliS is about half the size of maliXL, the shift in $\overline{x}$ is proportionally inversely dependent on *s*. Accordingly, both the averaged DEP force that acts on a particle and $\overline{x}$ are proportionally dependent on *s*^−1^∆*U*^2^.

An increase in throughput by 5 orders of magnitude compared to microfluidic applications is a significant step that highlights the potential to increase the throughput of DEP particle trapping by using this or similar porous ceramic filters. To some extent, the enhanced separation efficiency can be explained by a roughly 700-fold increase in cross section (230 mm^2^) the filter provides over the microchannels (0.336 mm^2^) used by Pesch et al. [17]. This means that in principle, a 700-fold increase of volumetric flow rate should be possible when increasing the cross section of the microchannels (e.g., by numbering up). This, however, explains only 3 of the actual 5 orders of magnitude difference. Additionally, the separation efficiency is influenced by the length and especially the tortuosity of the filter that determines the average particle residence time in the filter. The porous filter is approximately twice as long as the microchannels used by Pesch et al. [17] which just explains a factor of 2. We conclude that a main reason for the 2 remaining orders of improved trapping efficiency compared to the regular microstructure is the tortuous flow conditions in the porous filter. Since the structure is very inhomogeneous (providing a broad pore size and pore window size distribution and many sharp corners), strong fluid mixing is expected, resulting in an increased particle transport to the trapping zones. Furthermore, in such a randomized structure, it is likely that particles have to pass very small pore windows which provide ideal trapping conditions (high electric field gradients and low flow velocities). To underline the beneficial trapping conditions in the porous filter, we compare the separation against a packed bed of glass beads as used by, i.a., Suehiro et al. [21] (glass bead diameter 200 μm). For a packed bed (of the same dimensions as our ceramic filter) of glass beads with diameter *d*_sphere_ = 350 μm, $\overline{x}$ is more than 1 order of magnitude higher compared to results obtained with maliS (Fig. 4). The hydraulic pore diameter of a packed bed of uniform spheres (*d*_sphere_ = 350 μm) is calculated as *d*_h_ = 2/3 × *d*_sphere_Φ / (1 − Φ) = 156 μm, assuming a porosity of Φ = 0.4.

### Positive DEP and negative DEP filtration for electrical conductivity-selective particle separation

Compared to experiments in microchannels, it is not possible to directly observe particles and their behavior in the nontransparent ceramic filter. Instead, we used the fact that the DEP force is linearly dependent on the particle polarizability, represented by Re[*K*], which, at low frequencies, only depends on conductivities (Eq. (3)), to investigate their behaviors. Re[*K*] becomes 1 when the particle is much more conductive than the surrounding medium *σ*_P_ ≫ *σ*_m_, 0 for *σ*_P_ = *σ*_m_, and − 0.5 for *σ*_P_ ≪ *σ*_m_. If the DEP force is indeed driving particle trapping, the trapping efficiency is expected to follow the same or at least a similar trend as Re[*K*] and have a characteristic minimum at *σ*_P_ = *σ*_m_.

To show this, investigations were done with the ceramic filter maliS using 4.5-μm PS particles in an aqueous suspension. The conductivity of the suspension was stepwise increased around the particle conductivity (that we estimated to be between 1.7 × 10^−4^ and 17 × 10^−4^ S m^−1^; ESM, section E) from 1.2 × 10^−4^ to 22 × 10^−4^ S m^−1^. For reference, a similar experiment was also performed with highly conductive graphite particles (*σ*_graphite_ = 33 × 10^2^ S m^−1^ to 3 × 10^5^ S m^−1^) with average size of 3 μm.

**Fig. 5:**
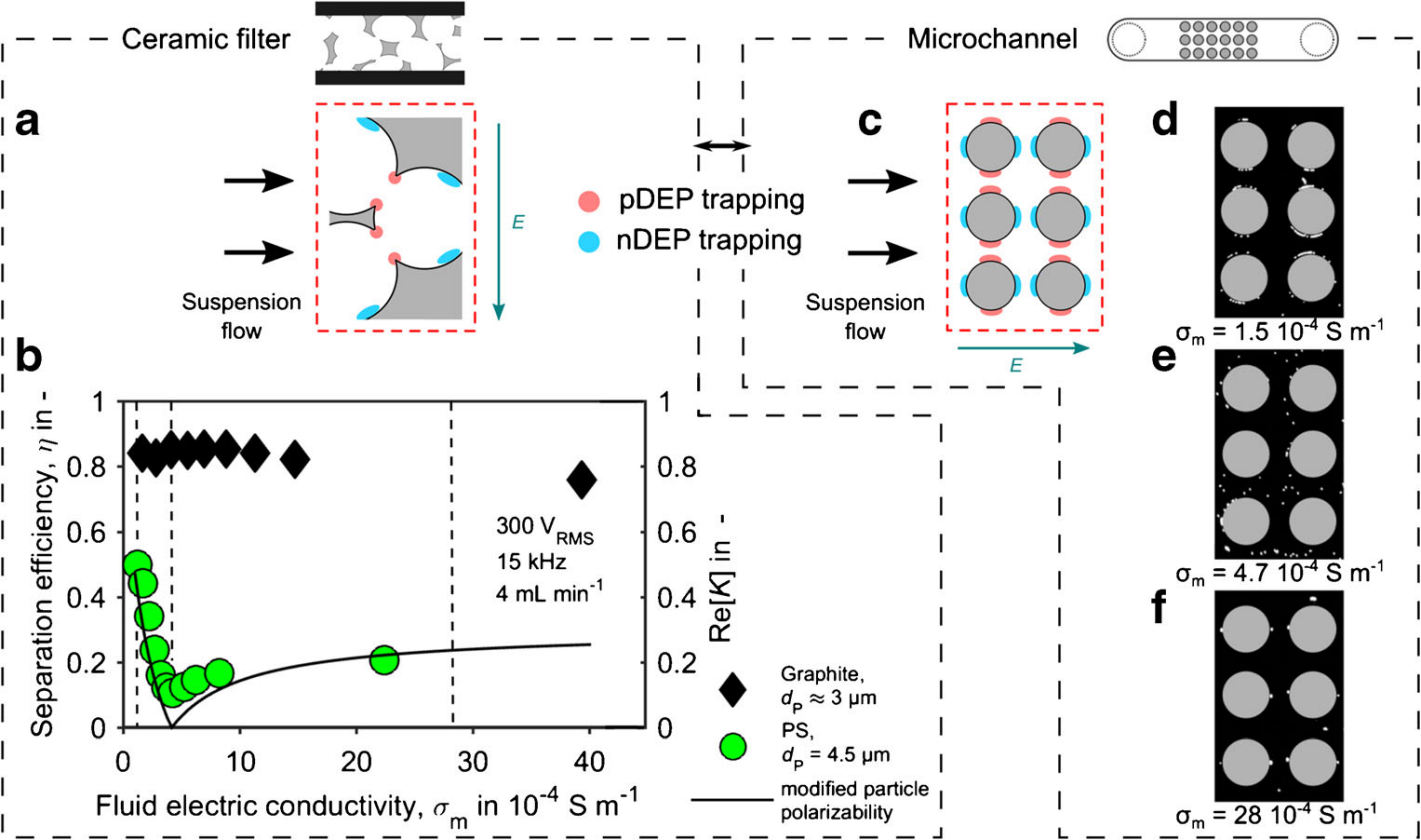
Particle trapping by pDEP and nDEP. A schematic of pDEP and nDEP particle trapping mechanisms at an exemplary pore window (**a**). Separation efficiency of PS and graphite particles in the ceramic filter maliS, each separately (not mixed) suspended in aqueous KCl solution, as a function of the fluid electric conductivity (**b**). The line represents the particle polarizability (Re[*K*]) for a particle with a conductivity of 4.2 μS cm^−1^. It shows the absolute value of Re[*K*] after Eq. (3) with the modification that negative values for Re[*K*] were multiplied by a fitting factor of 0.6. The results show the potential of DEP filtration to separate particles of different conductivities. Supporting experiments in microchannels were done to show that particles indeed experience pDEP, nDEP, or no DEP. A schematic of the trapping zones for pDEP and nDEP in the microchannels is shown on the right (**c**). The microscopy images (**d**–**f**) illustrate that particles show pDEP, no DEP, and nDEP at fluid electric conductivities of 1.5 × 10^−4^ S m^−1^, 4.2 × 10^−4^ S m^−1^, and 28 × 10^−4^ S m^−1^, respectively

As expected, PS particles were most efficiently trapped at the lowest investigated fluid electrical conductivity (*σ* = 1.2 × 10^−4^ S m^−1^) with the separation efficiency (*η*) being around 0.5 (Fig. 5b). With increasing fluid electrical conductivity, the separation efficiency fell to a minimum of *η* = 0.1 at about *σ* = 4.2 × 10^−4^ S m^−1^ and subsequently increased again to about *η* = 0.2. Graphite particles showed a higher separation efficiency of about *η* = 0.83 that was only very slightly dependent on *σ* and decreased slowly to *η* = 0.76 at *σ* = 40 × 10^−4^ S m^−1^.

The results in Fig. 5 reflect the theoretical predictions. Particles that are more conductive than the suspension experience pDEP. They are consequently attracted by the electric field maxima at the filter wall [34] where they are trapped tightly and thus most efficiently (Fig. 5a, left). PS particles show a minimum in separation efficiency at about 4.2 × 10^−4^ S m^−1^, which is in a plausible range for their conductivity [33, 35]. At higher medium electric conductivity, the PS particles experience nDEP that rejects the particles from the electric field maxima (Fig. 5a, right). In this case, particles can still be retained, since they get trapped at field minima in the filter and are rejected from the pore windows (where the electric field strength is high) and eventually cannot follow through with the fluid flow. However, nDEP trapping is expected to be less effective for three reasons.

1. As evident from the negative bound of Re[*K*] of − 0.5 compared to the positive bound 1, nDEP trapping can only be half as strong as pDEP trapping.
2. When experiencing nDEP, the majority of the particles are not tightly trapped at the walls but in the fluid where they are strongly affected by the fluid drag.
3. Simulations on microchannels and pillar arrays show that the gradient at field minima is by several orders of magnitude lower than the gradient at field maxima, indicating less holding forces for nDEP trapping.

To verify that the PS particles show pDEP, no DEP, and nDEP according to our interpretation, we did additional experiments with the same suspension at selected medium conductivities in microchannels. The microchannels are the same as used in our previous study [17]; see ESM for a comprehensive description of the experiments and channels. The different trapping zones that occur in the microchannels are visualized in Fig. 5 c. Our observations (Fig. 5d) match our interpretations very well: Indeed, at the low conductivity of 1.5 × 10^−4^ S m^−1^, the PS particles experience pDEP. At 4.2 × 10^−4^ S m^−1^, the observed minimum in the filtration experiments, particles are randomly dispersed in the suspension and appear not to be affected by DEP (Fig. 5e). At higher medium conductivities (28.1 × 10^−4^ S m^−1^), PS particles assemble at nDEP trapping zones where the electric field is lowest (Fig. 5f).

The separation results become even clearer when we compare the separation efficiency to the particle polarizability. A calculation of the absolute value of the particle polarizability represented by Re[*K*] (Eq. (3)) for an electric conductivity of 4.2 × 10^−4^ S m^−1^ matches the trend of the PS particles (black line in Fig. 5a). The data is rescaled by taking the absolute of Re[*K*] and by rescaling all negative values by a factor of 0.6. Employing such a (purely observational) rescaling factor for the negative part of Re[*K*] is justified as nDEP trapping is always less efficient (by an unknown factor) compared to pDEP trapping as described above. To further prove our interpretations, we compare the results against separation efficiencies obtained using graphite particles. They are much more conductive than the fluid and are thus experiencing pDEP throughout all tested conductivities (Fig. 5b, black symbols). A minute decrease of the separation efficiency at higher fluid conductivities can be observed and is ascribed to thermal and electrothermal effects that are likely to interfere with the DEP trapping process [27, 36].

We interpret these results as a direct proof that indeed dielectrophoresis is the main driving force behind the observed particle retention. Further, the results indicate that DEP filtration can be applied for electric conductivity-selective particle trapping. For completeness, the mechanical trapping was 8% for the 4.5-μm PS particles and 22% for the graphite particles.

### Filter capacity and recovery rate

**Fig. 6:**
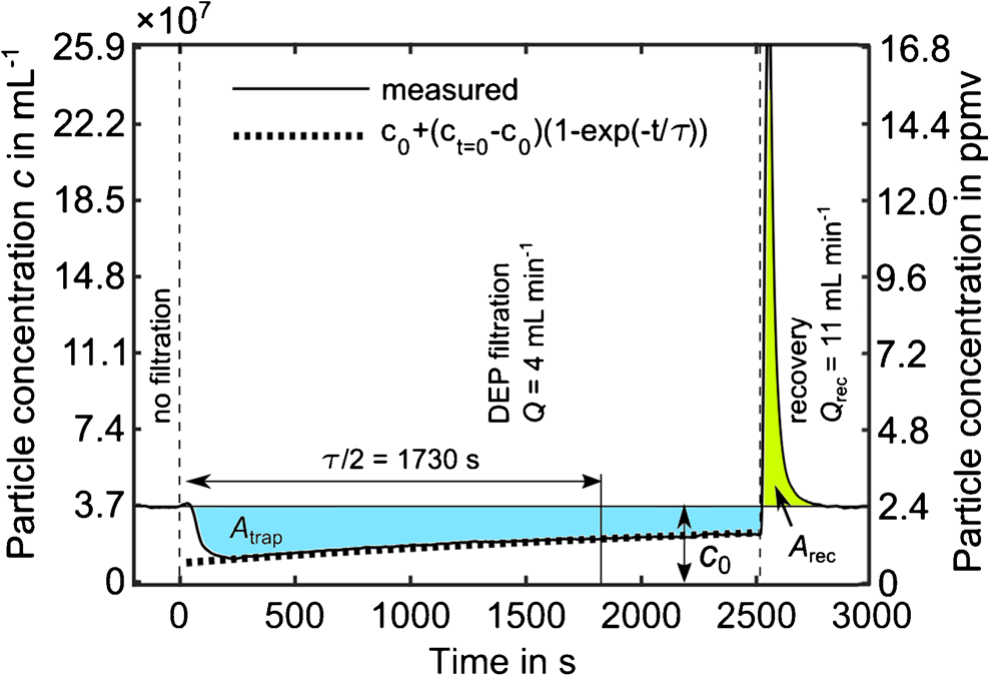
Long-term filtration experiments to determine filter capacity and recovery. Concentration of 500-nm PS particles in the suspension at the filter outlet during DEP filtration and subsequent particle recovery. The particle concentration in the suspension entering the filter is *c*_0_ = 3.7 × 10^7^ mL^−1^, corresponding to 2.4 × 10^−6^ vol/vol. The areas *A*_trap_ (blue) and *A*_rec_ (green) multiplied by the related flow rates during that times represent the normalized number of particles that was trapped and recovered. The volumetric flow (*Q*) during DEP filtration was 4 mL min^−1^ and was increased to 11 mL min^−1^ during recovery. The half characteristic relaxation time *τ*/2 is 1730 s

To investigate both filter capacity and particle recovery, we investigated the retention of 500-nm PS particles at a fluid electric conductivity of 1.5 × 10^−4^ S m^−1^. Compared to the previous experiments, DEP filtration was applied for a much longer time and the particle concentration in the suspension was increased to *c*_0_ = 3.7 × 10^7^ mL^−1^, to trap a sufficient number of particles for capacity investigations. Figure 6 shows the particle concentration in the suspension at the filter outlet over a period of ca. 45 min. The concentration of particles exiting the filter increases (and thus, the separation efficiency decreases) during DEP separation with an increasing number of particles already retained in the filter (Fig. 6). About 2.55 × 10^9^ particles, corresponding to 0.048‰ of the void volume, could be trapped until the separation efficiency decreased to 60.6% of the initial (i.e., maximum) trapping efficiency. The function *c*(*t*) = *c*_0_ − (*c*_start_ − *c*_0_)(1 − exp(−*t*/*τ*)), with *c*_start_ = *c*_0_ + (*c*_min_ − *c*_0_)/(1 − exp(−*t*_min_/*τ*)) and *t*_min_, the time when the particle concentration reaches its minimum, describes the particle concentration well. The results show that high particle concentrations and long filtration times are necessary to clog the filter, since even at increased particle concentration in our long-term experiment, the particle volume was about a factor of 10^4^ smaller than the porous volume of the filter.

In three long-term experiments, we found that 65% to 75% of the formerly trapped particles could be recovered immediately after switching of the electric field by flushing with the same suspension at 11 mL min^−1^. Considering all experiments of this study (including graphite), the recovery rate was between 40 and 100%. These relatively large deviations in recovery rate illustrate that the process is very sensitive to the filtration conditions such as the filter history (amount of permanently retained particles from previous filtration, etc.). It depends on the interaction between filter and particles due to electrostatic and van der Waals forces [30, 31] as well as wedging (retention in pore throats too small to pass) [32] effects that are influenced by the suspension (pH value and ionic strength) and the particle and filter surface (zeta potential, roughness, loading with already trapped particles, etc.). Investigation of these dependencies goes beyond the scope of this study. However, we repeated the long-term experiments under the same conditions but changed the fluid pH value during recovery to 8.5 (by adding KOH) which is above the isoelectric points of PS and mullite that have their isoelectric points at pH < 3 and pH = 7 [37], respectively. This led to negative zeta potentials of particles and filter and repulsive electrostatic forces between them, and the recovery rates increased substantially to values between 86% and 92%. That shows that the majority of the particles can be recovered in a very concentrated form simply by flushing the filter. The importance of the pH value was also observed regarding the mechanical retention of particles. When no electric field was applied, we found that the mechanical trapping of 4.5-μm PS particles was 8% at pH = 5.7 while it decreased to < 2% at pH = 8.5.

Potentially, particle recovery could significantly be improved when the DEP force is inverted for recovery so that particles experience nDEP and are repelled from pDEP trapping zones. According to Eq. (2), for particles of lower relative permittivity (*ε*_P_) than the suspension (*ε*_M_) (which is the case for most particles in aqueous suspension due to water’s high relative permittivity of 80), this can be achieved by increasing the electric field frequency above the Maxwell-Wagner relaxation frequency: *f*_MW_ = (*σ*_P_ + 2*σ*_M_) / (2*π* (*ε*_P_ + 2*ε*_M_)) [14].

## Conclusion

Classical dielectrophoretic approaches for particle separation show high selectivity and versatility but are not able to process larger amounts of liquid as required for many industrial processes like scrap recovery or in (bio-)analytical processes (for example in the detection of circulating cancer cells). This study shows that DEP filtration is capable to overcome the throughput gap between microscale and preparative or even industrial-scale applications. For the first time, we showed selective trapping and high recovery of sub-micron particles at throughputs in the mL min^−1^ range. We believe this to be a major step on the path towards industrial-scale applications of DEP separators. Achieving throughputs of small industrial scale is possible by a simple increase of the filter cross section. According to the test setup, a throughput of 1 L min^−1^ could be processed with a filter cross section of 0.022 m^2^ and 50 L min^−1^ with 1 m^2^, respectively. The presented results give further insights how the process can be adjusted by key parameters such as voltage, volumetric flow, and the structural dimensions of the filter.

An interesting next step would be to investigate selective DEP separation from mixtures of two different particles. A major limitation today is caused by Joule heating (heating due to electric current) that can lead to boiling or might, for example, cause degeneration/particle loss in biological applications. Consequently, the suspensions electrical conductivity (that directly correlates with the generated heat) can only be increased to a critical value that depends on how warm the suspension may become. To find ways to reduce the generated heat or how to lead it out of the process will be important. We would like to note in passing that using irregular filter structures does not allow filtration of strongly sedimenting particles because they cannot follow the suspension through the filter but sediment in regions of low fluid velocities. For such cases, filter media with convex solid structures with smooth surfaces like glass beads can be beneficial.

DEP filtration is still in its early steps and needs to be researched further before we can use its full potential. We think, however, that DEP filtration provides unique properties that can be used to reduce costs of a variety of existing separation processes or to make new and complex separation processes possible (such as material- and morphology-selective separation that have been achieved in microscale devices). This study shows that DEP filtration can play a major role in purely DEP-driven multistep separation processes or as an additional (preparative) tool in combination with other separation techniques. Low costs and remarkable process simplicity make DEP filtration processes attractive for further research and development of test devices.

## Electronic supplementary material

ESM 1 (PDF 1.14 mb)

## Data Availability

All data associated to this study is available from the authors upon reasonable request.
