# Supplementary material for: High-throughput dielectrophoretic filtration of sub-micron and micro particles in macroscopic porous materials
Source: Anal Bioanal Chem. 2020 Mar 21;412(16):3903–14. doi: 10.1007/s00216-020-02557-0 (PMC7235068; doi:10.1007/s00216-020-02557-0)
Supplement: Supplementary file 1 — (PDF 1.14 mb) [file 216_2020_2557_MOESM1_ESM.pdf]

Analytical and Bioanalytical Chemistry

Electronic Supplementary Material

High-throughput dielectrophoretic filtration of sub-micron and micro particles in macroscopic porous materials

Malte Lorenz, Daniel Malangré, Fei Du, Michael Baune, Jorg Thöming, Georg R. Pesch

A: FABRICATION OF OPEN POROUS ALUMINA-MULLITE FILTERS

The open porous ceramics were produced via direct foaming of a highly loaded particulate alumina mullite suspension. After frothing the ceramic slurry by incorporation of air the foam can be cast in various forms and self-solidifies while drying. The dried green foam samples are reaction sintered at 1650°C to obtain their ceramic properties. After cooling down the porous ceramics can be easily sawed and shaped to any form with conventional tools.

The low air content in the foam, in contrast to polyhedral foams, caused the bubble like structure and the perfectly spherical cell geometry. Cell opening (window formation) in the foam took place in the first stadium of drying after a certain amount of water is evaporated and before the structure becomes rigid.

Pore and pore window sizes are influenced by process parameters and the initial recipe of the slurry. The duration of foaming and the rotational speed of the impeller while frothing have a strong impact on the cell size. Other Parameters like the air to suspension ratio of the foam and the solid load in the suspension as well as the grain size and form of the particles that are used influence the stability of the foam and also affect the size of the pore windows. Porous ceramic foams with no interconnected pores (closed cell foams that are impermeable) as well as highly interconnected pores (open cell foams with a high permeability) and everything in between can be manufactured with our production method.

The successful usage of these porous ceramic foams as a filter material shows that there are far more applications than the initial one, sound absorption under high temperature.

The production procedure and the ceramic foams are patented (Deutsche Patentanmeldung Nr. 10 2018 106 260.5).

B: PORE DIAMETER AND PORE WINDOW DIAMETER FROM CT DATA

CT image stacks (920 to 1016 images of 1004x1024 pixel) of the alumina filter structures were obtained with a CT scanner phoenix v|tome|x m research edition (General Electric) funded by MAPEX (Center for Materials and Processes, University of Bremen, Germany). All structures were scanned with 6 µm voxel edge length so that the observed volume had was a cube with 6 mm edge length and contained at least 4300 pores and 5600 pore windows. The image stacks (exemplary image from the stack in Figure S1a) were subsequently binarized (filter material = 0, porous volume = 1) with ImageJ by using the ImageJ integrated median filter with a radius of 2 pixels and removing noise and very small pores of 3 pixels in diameter (ImageJ, Process, Noise, Remove Outliners…) (Figure S1b). Subsequently the DIPimage package version 2.9 was used in MATLAB to generate a 3d structure from the stack data and segregate the resulting porous volume into pores by using the watershed algorithm on the Euclidian distance transformed image stack (Figure S1c). We accounted only for pores that were completely present in the observed volume (pores connected to the frame of the image stack were removed). For each pore we calculated the volume equivalent sphere diameter and determined the volume weighed median pore diameter $d_{\mathrm{p},3}$ representative for the pore size distribution. The area equivalent pore window diameter of each surface that separated two pores (black lines between pores in Figure S1c) was also determined. Subsequently the area weighed median diameter of the pore window diameter $d_{\mathrm{w},2}$ distribution was calculated. The hydraulic pore diameter was calculated by $d_{\mathrm{h}} = \frac{4V}{S}$, with the total surface area of the ceramic S and the void volume V.

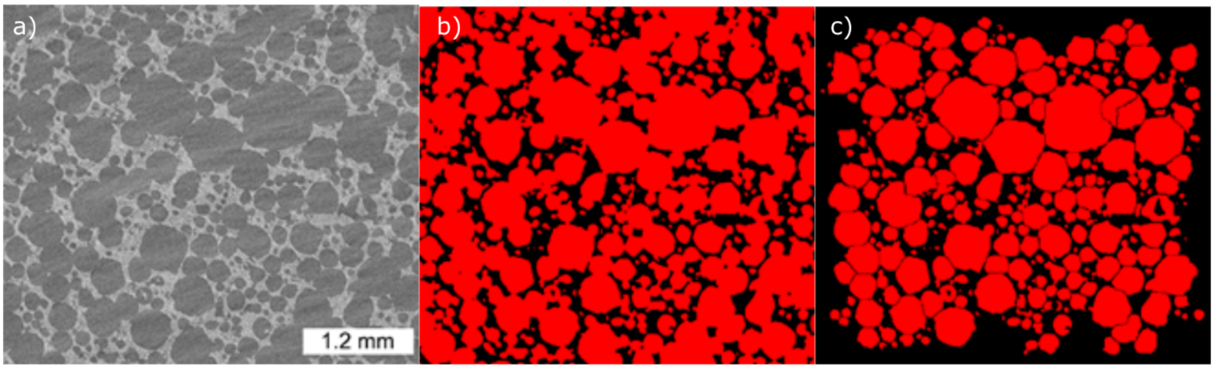

Fig. S1 The images show exemplary the progression from CT images to distinguished spherical pores for the ceramic maliM. We started with the unprocessed CT image (8-bit) (a). The image was then smoothened and binarized into solid and porous regions (b). Segmentation into spherical pores was done by cutting the pores at constrictions (3d watershed algorithm) (c)

C: COMSOL CALCULATION ON THE IMPACT OF THE FILTERS STRURAL SIZE ON $\nabla|\mathbf{E}_{\mathrm{RMS}}|^2$

We used the COMSOL Multiphysics® software (1) to model the electric field at different structural sizes and applied voltages. We assumed spherical pores with diameter d_P and pore windows of $d_W = 1/3\ d_P$. The electric field was calculated in water (relative permittivity $\varepsilon_W = 78$) that fills the void volume of the filter structure. Since the filter has a much lower permittivity (ε_F = 3-5) than water we assumed it to be electrically insulating ($\varepsilon = 0$). The voltage was applied over a distance of 565.7 µm at two planes that cut the middle of a pore and are aligned normalized to the symmetry axis through the middle of the pore window (Figure S2). An applied voltage of 21.2 V agrees to a global electric field strength of 37.5 kV m^{-1} that was generated at 300 V_{RMS} in our filtration setup. The distribution of $\nabla|\mathbf{E}_{\mathrm{RMS}}|^2$ for two exemplary calculations on the axial section are shown in Figure S2. The electric field was calculated for three cases that are presented in Table S1. The volume averaged $\nabla|\mathbf{E}_{\mathrm{RMS}}|^2$ scales with the applied voltage squared and inverse to the structural size.

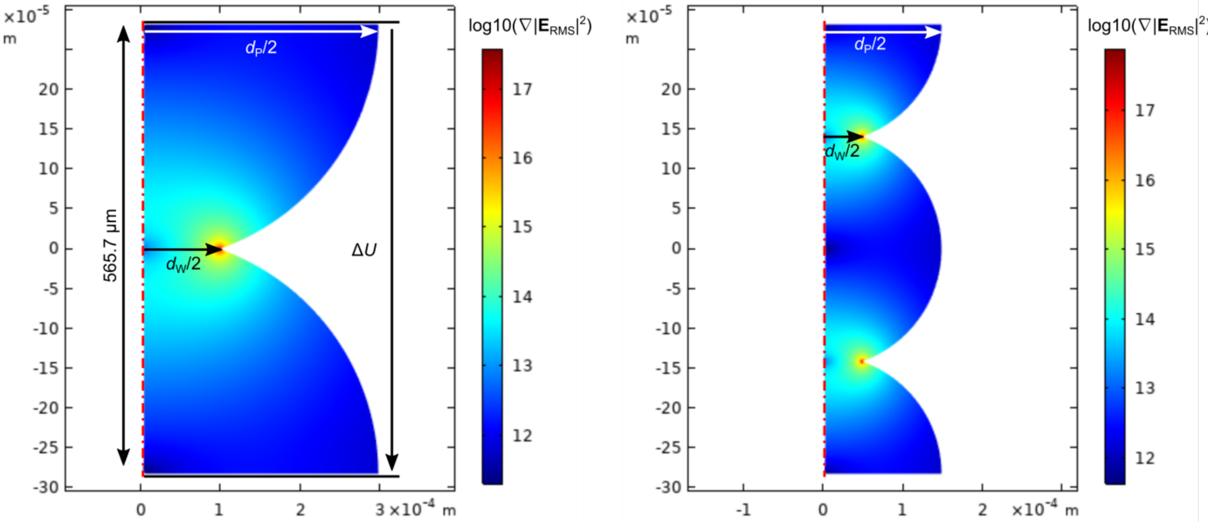

Fig. S2 Calculated logarithmic gradient of the squared electric field intensity for (left) d_P = 600 µm and d_W = 200 µm and (right) d_P = 300 µm, d_W = 100 µm. Shown is the axial section (axis, red dot dash line)

Table S1 Parameters for COMSOL calculation and the resulting volume averaged $\nabla|\mathbf{E}_{\mathrm{RMS}}|^2$

pore diameter d_P in µm	pore window diameter d_W in µm	applied voltage ΔU in V	volume averaged $|\mathbf{E}_{\mathrm{RMS}}|^2$ in $V^2\,m^{-3}$
600	200	21.2	3.35×10^{13}
300	100	21.2	6.7×10^{13}
600	200	42.4	13.4×10^{13}

D. EXPERIMENTS IN MICROCHANNELS FOR OBSERVATION OF pDEP AND nDEP PARTICLE TRAPPING

i. Microfluidic device design and fabrication

Microchannel experiments were performed with the same kind of PDMS microchannels that we used in a previous study (2). The procedure for producing microfluidic devices with polydimethylsiloxane (Sylgard 184, Dow Corning Corporation) is well established (3). First, a master mold was created using photo lithography with silica wafers and SU8. The photo mask that was used was created from a two-dimensional vector graphic (generated with AutoCAD, Autodesk). They were produced (CAD/Art Services Inc.) with a resolution of 20000 dpi. The smallest feature size is limited to 10 µm according to CAD/Art Services. A schematic of the microfluidic array is given in Figure S3. We used channels with post diameters of h_s = 262 µm and spacing of d = 100 µm. The array of posts had a length of 8.5 mm. In front and at the end of the channel half-elliptic posts are employed to avoid clogging of the channel due to any dust that might have been entrapped in the channel during production. The open channel between inlet and outlet and the array contains triangular support structures to prevent the channel from collapsing. To make the peel-off after curing easier, the SU8 negative was preconditioned with Trichloro(1H, 1H, 2H, 2H-perfluorooctyl)silane in an evacuated desiccator for a minimum duration of 60 min prior to soft lithography. Sylgard was mixed in a ratio of 10:1 (polymer to curing agent), degassed, and poured on the preconditioned SU8/Silica negative. Channels were cured at 140 °C for 15 min. Subsequently, the PDMS designs were sliced to the size of microscope slides (three channels per slide), and holes for the connection of PTFE tubing (ID/OD 300 µm/1.6 mm, Kinesis) were punched with 1.5 mm biopsy punches (World Precision Instruments Germany GmbH). For the electrodes (500 µm platinum wire) 0.5 mm diameter holes were punched with also with biopsy punches (also World Precision Instruments). PDMS-covered glass slides were prepared by spin coating isopropanol cleaned microscope slides with uncured PDMS at 3000 RPM for a duration of 1:30 minutes. The glass slides were subsequently cured at 70 °C for 60 min. Both sides of the channel were activated by exposing them to a low-pressure air plasma for 1:30 minutes. They were subsequently bonded by gently pressing them together. The channels in which all internal sides were PDMS had a final height of 120–130 µm (depending on the wafer used) and a width of 2.8 mm.

ii. Experimental procedure

For experiments we used the 4.5 µm particles (Polyscience Fluoresbrite, YG Carboxylate Microspheres 4.5 µm) that we used in the porous ceramic filters suspended in Milli-Q water but at a concentration of 4.2×10^5 particle cm^{-3}. The conductivity of the suspension was adjusted by adding KCl. A sinusoidal ac electric field (2000 V_{pp}, 15 kHz) was applied across

the array using two platinum wires connected to an ac high-voltage source (Trek PZD2000A in combination with a Rigol DG4000 arbitrary waveform generator). The spacing between the electrodes was 1 cm leading to an electric field strength of 20 $kV_{pp}\ m^{-1}$. Fluid was pumped into the channel using a syringe pump (kdScien- tific Legato 270 loaded with 3 mL PlastiPak disposable syringes). Prior to each experiment the device was flushed with the particle suspension for 30 s at 25 mL min^{-1}. For experiments we applied a flow rate of Q = 0.7 mL min^{-1}. To observe the particle behaviour, we used an inversed microscope Eclipse Ts2R-FL (Nikon) equipped with a 10 × Ph1 ADL (Nikon) objective glass and recorded with a DS-Fi3 (Nikon) camera and a C-LED470 filter set (Nikon, EX 470/40, DM 500, BA 534/55).

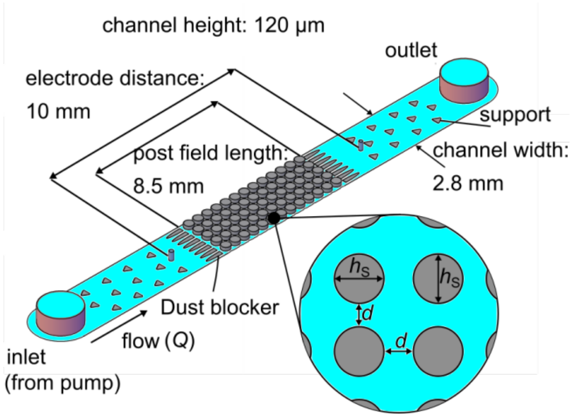

Fig. S3 Scheme of the microchannel that was used for observation of positive and negative DEP particle trapping. The microchannels have a height of 120 µm and a width of 2.8 mm. An array of insulating posts with cross-sectional diameter h_s

E. CALCULATING THE ELECTRIC CONDUCTIVITY OF PS PARTICLES

For our investigations on electrical conductivity selective particle trapping, it was important to approximate the electrical conductivity of the particles. In general, the particle conductivity can be assumed as a function of its bulk conductivity σ_b, its surface conductance K_s, and its radius a (4):

$$\sigma_p = \sigma_b + \frac{2K_s}{a}.$$

Since polystyrene is an electrically isolating material the bulk conductivity of a polystyrene particle is negligible and their conductivity is dependent on the double layer that exists at the interface between particle and its surrounding medium. At medium conductivities below 10^{-2} S m^{-1} and particle size equal or above 1 µm diameter, the PS particles conductivity is dominated by the Stern layer conductance which is mainly material dependent and can be assumed as independent of the medium conductivity (5). With the surface conductance of PS particles which is usually about 1 nS (6,7), the particle conductivity of a 4.5 µm particle is $\sigma_{p,PS} = \frac{2K_s}{a} = 8.8 \times 10^{-4} S\ m^{-1}$. However, the PS particle conductivity depends a lot on the specific particle and especially its surface (8). Surface conductivities between 0.2 nS and 2 nS have been reported (5,7) resulting in a wide range of conductivities between 1.7×10^{-4} S m^{-1} and to 17×10^{-4} S m^{-1}.

References

1. COMSOL Multiphysics® v. 5.4 [Internet]. Stockholm, Sweden: COMSOL AB;

2. Pesch GR, Lorenz M, Sachdev S, Salameh S, Du F, Baune M, et al. Bridging the scales in high-throughput dielectrophoretic (bio-)particle separation in porous media. Sci Rep. 2018;8(1):10480. doi: 10.1038/s41598-018-28735-w

3. Duffy DC, McDonald JC, Schueller OJA, Whitesides GM. Rapid Prototyping of Microfluidic Systems in Poly(dimethylsiloxane). Anal Chem. 1998;70(23):4974–84. doi: 10.1021/ac980656z

4. O'Konski CT. Electric properties of macromolecules. V. Theory of ionic polarization in polyelectrolytes. J Phys Chem. 1960;64(5):605–19. doi: 10.1021/j100834a023

5. Ermolina I, Morgan H. The electrokinetic properties of latex particles: comparison of electrophoresis and dielectrophoresis. J Colloid Interface Sci. 2005;285(1):419–28. doi: 10.1016/j.jcis.2004.11.003

6. Schwarz G. On the low-frequency dielectric dispersion of colloidal particles in electrolyte solution. J Phys Chem. 1962;66(12):2636–42. doi: 10.1021/j100818a067

7. Arnold WM, Schwan HP, Zimmermann U. Surface conductance and other properties of latex particles measured by electrorotation. J Phys Chem. 1987;91(19):5093–8. doi: 10.1021/j100303a043

8. Chow RS, Takamura K. Effects of surface roughness (hairiness) of latex particles on their electrokinetic potentials. J Colloid Interface Sci. 1988;125(1):226–36. doi: 10.1016/0021-9797(88)90071-9
